# Supplementary material for: A Likelihood Approach for Real-Time Calibration of Stochastic Compartmental Epidemic Models
Source: PLoS Comput Biol. 2017 Jan 17;13(1):e1005257. doi: 10.1371/journal.pcbi.1005257 (PMC5240920; doi:10.1371/journal.pcbi.1005257)
Supplement: S1 File — (TAR.GZ) [file pcbi.1005257.s014.tar.gz › HSPH_Online-SI-Revision/EnKF07/n100-extreme/n100-extreme_EnKF_table8.pdf]

1	R0	{2.76052, 16.3913}	{2.32095, 10.159}	{{0.391957, 3.48141}, {3., 17.7364}}	{27.0276, 41.4047}	inf	3200.	6849.44	{832.847, 15 037.6}	171.296	0.448536	newly	1155.	5408.19	{535., 18 342.}	407.703	0.538005	Reff	0.245824	0.48883	{0.0528747, 1.40186}	134.791	ar	92 090.	169 025.	{150 269., 227 517.}	83.5429
2	R0	{2.18202, 15.1734}	{2.14685, 2.88876}	{{0.0137907, 0.255049}, {3., 6.92304}}	{51.9056, 80.9617}	inf	3327.	7400.96	{1256.14, 5099.58}	141.511	0.308379	newly	1337.	11 991.3	{2353., 14 829.}	804.978	0.73817	Reff	0.385257	0.593368	{0.00105769, 0.743104}	124.921	ar	84 264.	185 147.	{168 770., 246 354.}	119.723
3	R0	{2.57891, 12.5971}	{2.45502, 13.0457}	{{1.39691, 8.91549}, {3., 16.978}}	{26.3571, 21.799}	inf	1362.	5092.05	{1752.33, 4483.04}	276.343	0.411441	newly	609.	2623.87	{754., 3293.}	333.706	0.447441	Reff	0.262765	0.551832	{0.198594, 0.82959}	120.234	ar	90 302.	162 704.	{150 398., 196 070.}	80.1774
4	R0	{2.5856, 9.28508}	{1.9464, 7.81165}	{{0.84862, 3.0588}, {3., 11.2969}}	{41.0692, 37.2428}	inf	433.	8166.48	{2842.68, 10 204.9}	1786.42	1.06398	newly	254.	6553.83	{2061., 8887.}	2480.55	1.1825	Reff	0.254526	0.526962	{0.191148, 0.850718}	119.988	ar	90 312.	189 056.	{168 177., 242 086.}	109.336
5	R0	{2.4406, 12.1341}	{2.60829, 8.81423}	{{2.02073, 1.01341}, {3., 12.8024}}	{20.6338, 30.22}	inf	1891.	5431.77	{1777.38, 4950.99}	192.259	0.363872	newly	894.	8067.19	{937., 12 585.}	804.359	0.593715	Reff	0.297534	0.737003	{0.293559, 2.02071}	161.767	ar	88 597.	172 658.	{153 304., 237 447.}	94.8799
6	R0	{2.72155, 15.4094}	{2.30586, 7.03022}	{{0.70639, 0.705429}, {3., 17.9955}}	{27.9608, 60.0859}	inf	3044.	12 702.6	{3672.43, 11 619.5}	322.71	0.517515	newly	1132.	13 761.8	{2907., 22 639.}	1119.54	0.88944	Reff	0.252533	0.586586	{0.131342, 1.21965}	161.276	ar	91 682.	179 443.	{155 702., 218 999.}	95.723
7	R0	{2.63781, 14.3935}	{2.45909, 3.63897}	{{1.53467, 2.6828}, {3., 4.56514}}	{24.076, 74.7179}	inf	2374.	7404.04	{1781.82, 8893.14}	225.405	0.323858	newly	968.	9815.7	{2393., 10 037.}	920.397	0.735	Reff	0.252043	0.529724	{0.126653, 0.795847}	126.022	ar	91 192.	183 588.	{165 776., 198 120.}	101.321
8	R0	{2.82391, 16.104}	{2.31933, 7.18099}	{{0.530725, 0.872307}, {3., 17.1547}}	{25.85, 58.3711}	inf	3239.	12 544.1	{1280.12, 12 369.5}	306.373	0.569069	newly	1178.	12 116.2	{1373., 21 962.}	940.183	0.876134	Reff	0.232408	0.57061	{0.11162, 0.943384}	169.797	ar	92 664.	178 275.	{152 196., 208 973.}	92.3891
9	R0	{2.80645, 14.6457}	{2.31467, 3.26531}	{{0.684709, 3.01322}, {3., 4.0998}}	{26.3508, 77.7047}	inf	2875.	10 833.7	{549.428, 41 829.1}	311.209	0.541864	newly	1160.	13 679.1	{753., 47 812.}	1093.52	0.817079	Reff	0.229399	0.436139	{0.0435524, 0.784344}	118.451	ar	92 567.	185 933.	{155 776., 283 135.}	100.864
10	R0	{1.86213, 5.4271}	{2.23551, 4.95014}	{{1.39533, 0.738179}, {3., 17.397}}	{43.5595, 78.3471}	inf	95.	7655.37	{1200.71, 20 338.4}	7958.29	1.66978	newly	92.	9129.85	{1009., 10 110.}	9823.75	1.70427	Reff	0.460598	0.416654	{0.0826992, 0.591466}	49.1649	ar	75 362.	197 572.	{177 822., 235 944.}	162.164
11	R0	{2.27349, 14.6338}	{2.71431, 8.66695}	{{2.17515, 2.27483}, {3., 14.8368}}	{21.7255, 41.7762}	inf	2757.	5410.62	{401.599, 16 314.2}	163.02	0.408452	newly	1137.	7035.06	{278., 16 071.}	560.61	0.507357	Reff	0.351982	0.613431	{0.143267, 1.70696}	114.738	ar	85 805.	171 704.	{150 268., 230 512.}	100.11
12	R0	{2.77261, 14.1894}	{2.68698, 3.52052}	{{2.07935, 2.62139}, {3., 4.99662}}	{13.9142, 75.1891}	inf	2393.	5025.94	{753.006, 13 998.8}	132.52	0.329567	newly	959.	10 689.7	{933., 27 795.}	1022.39	0.769278	Reff	0.226855	0.704489	{0.252899, 2.07933}	214.323	ar	92 508.	193 244.	{165 715., 255 765.}	108.894
13	R0	{2.65402, 19.3935}	{2.14255, 6.79824}	{{1.12979, 0.442112}, {3., 16.7042}}	{33.3503, 64.9458}	inf	6091.	8286.98	{4432.58, 25 976.8}	86.8291	0.259019	newly	2008.	6498.53	{2253., 12 912.}	233.427	0.456297	Reff	0.292659	0.536489	{0.0431989, 1.14833}	121.774	ar	91 184.	190 619.	{170 727., 243 535.}	109.049
14	R0	{1.75297, 4.45354}	{2.76056, 10.0486}	{{2.20187, 0.82236}, {3., 13.348}}	{57.4791, 158.25}	inf	33.	10 086.9	{296.997, 7203.49}	30 468.1	1.97211	newly	37.	9475.5	{170., 15 956.}	25 513.7	1.81235	Reff	0.50468	0.96754	{0.582371, 2.20185}	95.4238	ar	71 263.	193 356.	{160 235., 256 061.}	171.327
15	R0	{1.99928, 7.1501}	{2.67418, 10.7961}	{{2.01266, 1.64439}, {3., 15.8228}}	{33.7571, 89.5039}	inf	273.	4191.72	{1351.54, 3174.03}	1435.43	1.01997	newly	197.	2997.01	{803., 4769.}	1421.32	0.970636	Reff	0.411872	0.594052	{0.124966, 1.25873}	95.8103	ar	79 602.	168 309.	{153 828., 219 745.}	111.439
16	R0	{2.67398, 10.3134}	{2.7109, 11.577}	{{2.00309, 3.38308}, {3., 16.0588}}	{15.9326, 48.2745}	inf	624.	5892.14	{2143.38, 4801.63}	847.579	0.829071	newly	342.	3933.08	{1033., 8005.}	1051.88	0.852368	Reff	0.237957	0.626083	{0.0550425, 2.00307}	217.624	ar	91 289.	178 252.	{160 708., 244 365.}	95.2613
17	R0	{2.44634, 16.3226}	{2.23662, 6.90039}	{{1.25952, 1.95372}, {3., 15.085}}	{33.9208, 57.7249}	inf	3997.	7155.07	{588.258, 26 270.8}	129.675	0.314721	newly	1461.	6763.61	{587., 10 863.}	382.647	0.496663	Reff	0.317486	0.53266	{0.114217, 1.02254}	98.3231	ar	88 710.	187 186.	{168 371., 223 132.}	111.009
18	R0	{2.02168, 10.1676}	{2.60213, 3.77092}	{{1.75005, 2.1814}, {3., 5.26701}}	{33.7479, 62.9124}	inf	1489.	3915.49	{1574.11, 11 240.7}	178.699	0.384417	newly	847.	5951.96	{1581., 22 316.}	614.982	0.650642	Reff	0.420772	0.464964	{0.104586, 0.946837}	68.004	ar	80 299.	176 533.	{158 741., 210 445.}	119.844
19	R0	{2.89969, 15.009}	{2.1991, 6.45265}	{{0.597324, 1.4509}, {3., 12.6938}}	{28.2428, 57.0082}	inf	2686.	9633.06	{1264.01, 16 501.1}	277.677	0.449845	newly	1026.	9621.75	{815., 24 001.}	851.36	0.762077	Reff	0.211938	0.507357	{0.0975184, 0.98035}	162.87	ar	93 349.	179 176.	{157 399., 242 955.}	91.9422
20	R0	{2.68933, 7.34928}	{2.54976, 6.39737}	{{1.90186, 4.32145}, {3., 9.26602}}	{18.821, 31.1712}	inf	136.	2048.69	{57.1221, 2035.41}	1427.94	0.825766	newly	80.	1994.03	{56., 1980.}	2407.29	0.969993	Reff	0.227975	0.620644	{0.240952, 1.90184}	177.767	ar	91 574.	188 088.	{171 107., 254 387.}	105.394
21	R0	{2.15352, 9.28074}	{2.64653, 9.54599}	{{2.01813, 0.870098}, {3., 12.965}}	{27.4197, 42.7266}	inf	838.	4428.63	{2015.2, 4329.96}	430.356	0.590699	newly	504.	4243.45	{1285., 10 387.}	743.831	0.682433	Reff	0.358733	0.798619	{0.351326, 2.01811}	128.918	ar	83 845.	170 962.	{153 121., 233 065.}	103.902
22	R0	{2.21953, 9.04387}	{1.99092, 5.65116}	{{0.223431, 0.396253}, {3., 16.0493}}	{54.3522, 80.1034}	inf	694.	8393.76	{1269.36, 18 436.5}	1111.85	0.805342	newly	414.	9194.28	{1337., 23 181.}	2121.29	0.977059	Reff	0.343938	0.300838	{0.0468533, 0.604223}	60.1593	ar	84 856.	182 091.	{165 511., 211 189.}	114.588
23	R0	{2.21987, 14.5347}	{2.74266, 3.15327}	{{2.28943, 0.0955231}, {3., 6.52271}}	{25.547, 78.3053}	inf	2885.	7060.17	{5456.5, 8118.28}	146.168	0.332809	newly	1192.	14 919.2	{3937., 45 070.}	1151.7	0.883362	Reff	0.366057	0.683457	{0.216157, 2.28941}	114.692	ar	85 067.	194 400.	{172 676., 262 826.}	128.525
24	R0	{2.84268, 17.9659}	{2.20144, 4.13972}	{{0.0233164, 1.9799}, {3., 6.27217}}	{30.3054, 76.9579}	inf	4541.	5051.04	{680.903, 4819.48}	49.6769	0.265334	newly	1477.	4975.41	{882., 6950.}	253.664	0.475597	Reff	0.242139	0.30273	{0.00786005, 0.650417}	81.9102	ar	92 836.	199 409.	{191 252., 205 229.}	114.797
25	R0	{2.52706, 10.3679}	{2.11054, 3.73506}	{{0.217858, 1.53735}, {3., 7.32452}}	{41.9158, 63.9748}	inf	746.	5280.3	{1582.54, 8500.81}	614.377	0.721351	newly	432.	5203.89	{1158., 9035.}	1109.93	0.929859	Reff	0.269107	0.205228	{0.026244, 0.468719}	70.2301	ar	89 623.	167 161.	{154 982., 207 347.}	86.5155
26	R0	{2.62835, 19.1088}	{2.44779, 4.84397}	{{1.89446, 0.55689}, {3., 12.2467}}	{21.3196, 74.6506}	inf	5501.	5454.19	{1041.48, 15 266.4}	79.7883	0.366072	newly	1758.	6117.53	{1339., 11 623.}	263.098	0.508686	Reff	0.292378	0.573014	{0.035456, 1.25692}	135.41	ar	90 908.	187 210.	{168 340., 239 192.}	105.933
27	R0	{2.76831, 19.4155}	{2.77187, 10.2339}	{{2.20771, 3.72782}, {3., 18.1002}}	{11.2539, 47.2899}	inf	4502.	15 707.1	{1380.76, 68 327.4}	291.854	0.526291	newly	1418.	12 024.8	{499., 25 127.}	775.666	0.739031	Reff	0.252857	0.643593	{0.0535254, 1.30962}	178.844	ar	92 175.	190 217.	{156 724., 266 341.}	106.365
28	R0	{2.96609, 16.8903}	{2.7334, 10.8329}	{{2.13999, 3.3886}, {3., 15.4224}}	{9.42285, 35.8634}	inf	3132.	5210.2	{606.082, 7095.04}	118.243	0.366454	newly	1123.	3756.03	{285., 7202.}	274.117	0.49819	Reff	0.198639	0.713194	{0.0183487, 2.13996}	295.311	ar	94 024.	173 024.	{150 078., 239 456.}	84.0209
29	R0	{2.17046, 14.39}	{2.10358, 4.39485}	{{0.0984261, 2.14102}, {3., 7.05284}}	{55.8244, 69.459}	inf	3337.	7893.09	{4854.48, 4854.48}	143.601	0.25514	newly	1397.	8973.4	{3082., 18 242.}	544.097	0.609153	Reff	0.390705	0.364326	{0.0189614, 0.767319}	66.3898	ar	84 001.	174 829.	{163 800., 196 984.}	108.127
30	R0	{2.88772, 12.4647}	{1.98994, 5.16279}	{{0.848906, 4.06585}, {3., 5.73462}}	{35.2109, 58.5808}	inf	1402.	2385.9	{1228.38, 1249.95}	96.985	0.16527	newly	651.	2525.19	{936., 1710.}	292.813	0.359913	Reff	0.203498	0.448708	{0.180051, 0.793957}	127.549	ar	93 273.	167 824.	{161 262., 165 984.}	79.9281
31	R0	{2.46831, 8.14308}	{2.47317, 8.4623}	{{1.90243, 5.69112}, {3., 9.46763}}	{22.2055, 10.892}	inf	300.	3883.43	{1315.88, 4718.19}	1194.48	0.919602	newly	189.	3432.36	{822., 3680.}	1716.06	0.986764	Reff	0.280894	0.711303	{0.126158, 1.90241}	171.066	ar	88 762.	180 014.	{160 304., 257 663.}	102.806
32	R0	{1.93236, 7.38987}	{2.22331, 10.9864}	{{0.636281, 8.01014}, {3., 12.6865}}	{57.9036, 48.7917}	inf	389.	4552.15	{874.409, 4988.84}	1070.34	0.806284	newly	288.	2783.22	{418., 3144.}	866.694	0.669346	Reff	0.437467	0.426564	{0.0489847, 0.637323}	70.3321	ar	77 654.	170 052.	{159 051., 182 570.}	118.987
33	R0	{2.82251, 17.6954}	{2.62151, 13.9695}	{{1.64762, 9.40313}, {3., 17.593}}	{16.1765, 22.1174}	inf	4866.	6930.	{783.03, 5991.94}	81.2488	0.298183	newly	1670.	3385.58	{315., 4150.}	138.228	0.39449	Reff	0.244147	0.669347	{0.140515, 1.64675}	189.336	ar	92 745.	170 839.	{147 790., 224 537.}	84.2033
34	R0	{1.87083, 12.3994}	{2.05081, 2.44207}	{{0.197665, 0.295561}, {3., 7.04176}}	{70.0502, 80.3049}	inf	3095.	2539.81	{1534.19, 2288.67}	55.9302	0.253348	newly	1528.	3654.84	{1256., 6077.}	149.031	0.309335	Reff	0.498595	0.403523	{0.0277522, 0.732987}	52.5426	ar	76 282.	162 258.	{156 661., 164 140.}	112.709
35	R0	{2.95779, 16.3003}	{2.61699, 13.7593}	{{1.67919, 9.51482}, {3., 17.017}}	{13.5485, 18.687}	inf	3818.	7481.87	{397.644, 6488.41}	123.77	0.447846	newly	1428.	3747.59	{171., 4358.}	189.359	0.544127	Reff	0.209086	0.655532	{0.195654, 1.2545}	223.706	ar	93 873.	168 729.	{145 517., 217 961.}	79.7418
36	R0	{2.18718, 14.261}	{2.0273, 7.23526}	{{0.0414071, 3.74158}, {3., 10.182}}	{54.9386, 49.2654}	inf	3033.	7292.66	{371.534, 24 140.9}	183.505	0.380804	newly	1295.	5240.63	{217., 12 701.}	340.988	0.549138	Reff	0.381269	0.421083	{0.0031139, 0.671121}	90.4298	ar	84 379.	170 310.	{149 860., 213 445.}	101.84
37	R0	{2.44912, 9.31118}	{2.37363, 4.93501}	{{1.35426, 2.3222}, {3., 11.0192}}	{30.074, 59.7362}	inf	559.	8342.41	{4196.13, 14 986.9}	1394.03	0.972638	newly	306.	9910.04	{1743., 18 174.}	3140.38	1.23905	Reff	0.289559	0.388156	{0.0136373, 0.664728}	89.3803	ar	88 442.	202 178.	{182 432., 272 936.}	128.6
38	R0	{2.05949, 5.72514}	{2.36836, 10.275}	{{1.42917, 2.7457}, {3., 13.9239}}	{38.9394, 102.47}	inf	81.	3601.28	{502.047, 3162.96}	4346.7	1.51851	newly	76.	3131.72	{242., 4991.}	4021.84	1.35316	Reff	0.380141	0.417166	{0.0777403, 0.571945}	63.619	ar	81 587.	182 546.	{174 752., 181 546.}	123.744
39	R0	{1.75505, 8.52404}	{2.45968, 4.13217}	{{1.53691, 2.54164}, {3., 4.73614}}	{49.2296, 51.5233}	inf	1029.	10 803.3	{4764.52, 19 431.4}	949.88	0.810706	newly	719.	11 960.4	{4500., 22 807.}	1563.47	1.01642	Reff	0.527357	0.517486	{0.243323, 0.861225}	36.631	ar	70 925.	183 876.	{163 227., 217 961.}	159.254
40	R0	{2.24724, 13.7861}	{2.12596, 3.41804}	{{0.257675, 1.71404}, {3., 8.0318}}	{47.5038, 75.2066}	inf	2420.	4431.81	{2181.01, 9097.67}	106.932	0.19092	newly	1045.	5278.74	{1289., 19 750.}	407.897	0.488672	Reff	0.360008	0.312161	{0.0392072, 0.439582}	64.2979	ar	85 257.	171 769.	{157 374., 211 393.}	101.472
41	R0	{1.77635, 8.62406}	{2.26505, 8.41569}	{{0.819472, 0.696734}, {3., 17.7325}}	{55.1513, 75.5722}	inf	882.	10 616.5	{2458.74, 12 748.6}	1107.28	0.862172	newly	608.	9541.75	{1520., 15 654.}	1473.54	0.920399	Reff	0.494927	0.496755	{0.0791535, 0.784}	52.8393	ar	72 941.	179 147.	{158 943., 207 752.}	145.605
42	R0	{1.80397, 5.4932}	{2.59909, 7.72232}	{{1.72352, 2.80461}, {3., 11.4965}}	{45.267, 66.0189}	inf	102.	8464.17	{3424.95, 7484.49}	8203.7	1.78817	newly	82.	7203.41	{1778., 11 290.}	8690.96	1.79573	Reff	0.48442	0.598648	{0.361704, 0.745565}	40.8715	ar	73 232.	201 821.	{182 280., 250 223.}	175.591
43	R0	{2.83096, 16.7077}	{2.60204, 5.07898}	{{1.97531, 0.566519}, {3., 10.9342}}	{15.0127, 69.601}	inf	3887.	4202.46	{547.981, 2257.7}	103.918	0.438246	newly	1377.	4800.02	{301., 12 630.}	282.999	0.489279	Reff	0.237546	0.477685	{0.0465802, 0.668526}	126.701	ar	92 740.	180 131.	{166 744., 227 933.}	94.2322
44	R0	{2.73678, 11.6021}	{2.12793, 4.34598}	{{0.90798, 2.51315}, {3., 5.33598}}	{33.0191, 62.5414}	inf	851.	4117.5	{806.674, 3287.7}	395.354	0.565939	newly	393.	5223.34	{575., 5282.}	1237.26	0.865446	Reff	0.225237	0.331249	{0.05185, 0.531844}	83.0036	ar	92 003.	185 882.	{172 031., 215 608.}	102.039
45	R0	{2.14235, 6.98601}	{2.57193, 11.448}	{{2.00448, 3.47704}, {3., 16.1912}}	{25.5861, 94.0895}	inf	172.	5874.39	{1010.04, 4870.39}	3316.27	1.26419	newly	135.	3715.94	{486., 5875.}	2653.79	1.14905	Reff	0.36707	0.639467	{0.159625, 2.00446}	115.117	ar	82 974.	180 722.	{162 699., 239 972.}	117.805
46	R0	{2.23409, 9.21289}	{2.68399, 9.54168}	{{2.0424, 1.11735}, {3., 12.8251}}	{25.801, 40.5801}	inf	866.	5252.99	{1852.77, 5143.09}	513.699	0.676612	newly	520.	4670.95	{904., 8067.}	800.66	0.689347	Reff	0.341659	0.530224	{0.113684, 0.806123}	103.399	ar	85 163.	168 471.	{151 037., 231 871.}	97.8223
47	R0	{2.12258, 8.15265}	{2.37726, 9.02372}	{{1.68128, 3.39227}, {3., 14.6601}}	{28.5122, 70.2429}	inf	433.	13 816.4	{1700.95, 12 145.2}	3090.86	1.31677	newly	280.	15 849.6	{1492., 31 474.}	5560.56	1.41804	Reff	0.375293	0.696658	{0.201466, 1.37965}	112.895	ar	82 690.	212 807.	{177 230., 288 007.}	157.355
48	R0	{2.71771, 10.4636}	{2.35618, 4.91828}	{{1.35627, 1.67377}, {3., 12.9979}}	{25.1438, 65.8926}	inf	924.	10 257.1	{5395.26, 24 123.9}	1014.57	0.851485	newly	498.	11 966.9	{3005., 13 621.}	2308.97	1.15178	Reff	0.232147	0.443972	{0.135843, 0.91378}	115.844	ar	91 716.	204 951.	{181 641., 265 225.}	123.462
49	R0	{2.44369, 6.66477}	{2.52345, 6.78655}	{{1.83768, 4.24368}, {3., 9.49677}}	{23.599, 38.1539}	inf	49.	3840.94	{555.919, 4009.5}	7738.66	1.71711	newly	43.	4631.94	{618., 5209.}	10 672.	1.76682	Reff	0.273693	0.735449	{0.0118824, 1.40755}	188.424	ar	88 827.	187 378.	{170 272., 216 337.}	110.947
50	R0	{2.77383, 17.9775}	{2.2688, 4.44137}	{{1.41031, 0.702645}, {3., 5.99081}}	{26.687, 75.2949}	inf	3843.	6483.52	{3490.9, 12 050.}	88.2501	0.176508	newly	1236.	6866.85	{2664., 20 230.}	458.625	0.571884	Reff	0.244319	0.373368	{0.111247, 0.573674}	90.6394	ar	92 334.	175 372.	{160 139., 219 582.}	89.9322
